# Neck Circumference and the Heart: Unveiling a New Anthropometric Marker for LVH Risk in Type 2 Diabetes—The TESEO Study

**DOI:** 10.1002/dmrr.70106

**Published:** 2025-11-14

**Authors:** Federica Barutta, Alessandro Andreis, Guglielmo Beccuti, Arianna Ferro, Martina Bollati, Stefania Bellini, Giulia Montesano, Giulio Mengozzi, Matteo Bellettini, Gaetano M. De Ferrari, Gianluca Alunni, Fabio Broglio, Gabriella Gruden

**Affiliations:** ^1^ Department of Medical Sciences University of Turin Turin Italy; ^2^ Advanced Cardiovascular Echocardiography Unit Cardiovascular and Thoracic Department Città della Salute e della Scienza di Torino University Hospital Turin Italy; ^3^ Division of Cardiology Città della Salute e della Scienza di Torino University Hospital Turin Italy

**Keywords:** left ventricular hypertrophy, neck circumference, neck circumference to height ratio, type 2 diabetes

## Abstract

**Aim:**

Left ventricular hypertrophy (LVH) is highly prevalent among individuals with type 2 diabetes (T2DM) and is a predictor of adverse cardiovascular outcomes. Visceral and ectopic fat accumulation contributes to cardiometabolic risk. Neck circumference (NC) and the neck circumference‐to‐height index (NCI) have emerged as potential markers of ectopic adiposity, yet their association with LVH remains unexplored. The aim of this study was to evaluate the relationship between NC and NCI with the presence of LVH in a contemporary cohort of T2DM patients.

**Materials and Methods:**

T2DM patients were consecutively enroled in the TESEO study. Participants underwent comprehensive clinical, biochemical, bioimpedance and echocardiographic evaluations. Multivariable logistic regression models were used to examine the independent associations between NC/NCI and LVH, adjusting for age, sex, mean arterial blood pressure, HbA1c, triglycerides, estimated glomerular filtration rate, urinary albumin‐to‐creatinine ratio and treatments.

**Results:**

29% of participants had LVH. NC and NCI were higher among individuals with LVH. After full adjustment, NC and NCI were independently associated with 26% and 57% higher odds of LVH, respectively. Stratified analyses revealed a stronger association in men (OR 1.65, 95% CI: 1.25–2.18) than in women (OR 1.49, 95% CI: 1.19–1.87). NC and NCI outperformed BMI and waist circumference in their association with LVH. ROC curve analyses confirmed that NCI has good discriminatory power for identifying individuals with and without LVH.

**Conclusions:**

NC and NCI are independently associated with LVH in T2DM patients and they may represent practical, non‐invasive markers to enhance cardiovascular risk stratification in clinical settings.

## 1 Introduction

Left ventricular hypertrophy (LVH) is a well‐established predictor of cardiovascular (CV) morbidity and mortality. It is highly prevalent among individuals with type 2 diabetes mellitus (T2DM) and is strongly associated with an increased risk of coronary artery disease (CAD), stroke, sudden cardiac death and heart failure (HF) in this population [1].

LVH often remains clinically silent in its early stages and is frequently undetected until severe structural and functional cardiac abnormalities have developed. Regression of LVH can lead to meaningful reductions in CV events and mortality among patients with T2DM. As such, early identification of individuals at high risk is essential for implementing timely preventive or therapeutic strategies aimed at reducing CV burden and improving clinical outcomes [2].

Among the pathophysiological mechanisms linking T2DM to LVH, hypertension remains the predominant driver; however, obesity also plays a significant role. In particular, excess visceral adipose tissue (VAT) contributes to the development of insulin resistance and chronic low‐grade systemic inflammation—two interrelated processes that are critically involved in the pathogenesis of LVH [3]. As a result, anthropometric markers, such as body mass index (BMI) and waist circumference (WC), have been extensively studied for their association with LVH [4, 5].

More recently, neck circumference (NC) has gained attention as a new simple, non‐invasive anthropometric marker. Neck adipose tissue belongs to the upper‐body subcutaneous fat (UBSF), but it is functionally similar to VAT [6, 7]. Unlike other anthropometric measures of VAT, NC is minimally affected by factors such as posture, breathing, recent food intake, transient abdominal distension or pregnancy. Combined with its clear anatomical landmarks, high reproducibility, and low measurement variability, NC is a particularly reliable anthropometric indicator.

Both NC and the neck circumference‐to‐height index (NCI) have been associated not only with other anthropometric parameters [8, 9, 10, 11, 12, 13, 14, 15], but also with dysmetabolic conditions, including metabolic syndrome and its components [11, 13, 16, 17, 18, 19, 20, 21, 22], non‐alcoholic fatty liver disease [23, 24] and diabetes mellitus [25, 26]. Furthermore, NC has also been associated with arterial stiffness [27, 28], carotid intima‐media thickness [29], clinical CV events [19, 30, 31] and all‐cause mortality [19, 32]. In a prospective study, NC was significantly associated with the risk of future CV events [30]. Similarly, in a large Chinese cohort of individuals at high CV risk, greater NC was independently associated with a higher incidence of both fatal and non‐fatal CV events, as well as all‐cause mortality [31].

However, data on the relationship between NC/NCI and LVH remain lacking, both in patients with T2DM and in the general population. Therefore, the aim of the present study was to evaluate the potential independent association between NC/NCI and LVH in a contemporary cohort of individuals with T2DM.

## 2 Methods

### 2.1 Study Population

**FIGURE 1:**
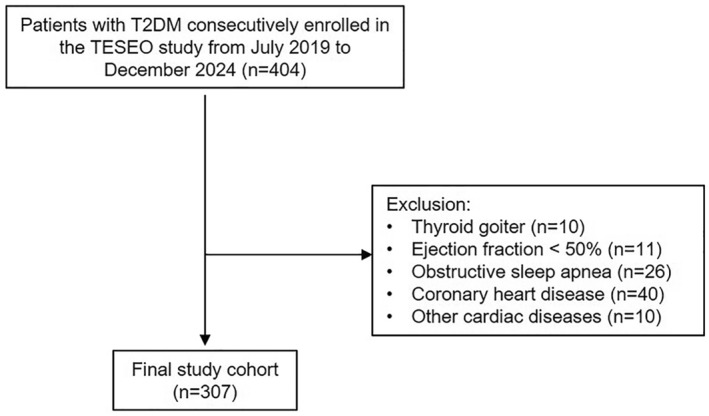
Flow chart of the study.

The study was conducted on patients with T2DM, who were prospectively and consecutively recruited between July 2019 and October 2024 as part of the TESEO cohort study on chronic complications of T2DM. Patients were eligible for inclusion if they met the following criteria: age between 18 and 80 years, first referral to the Unified Diabetes Centre at San Giovanni Antica Sede Hospital in Turin, and availability of echocardiographic and anthropometric data, including NC. Exclusion criteria were the presence of thyroid goitre/dysfunction, left ventricular ejection fraction (LVEF) < 50%, coronary artery disease (CAD), moderate‐to‐severe valvular heart disease, biological or mechanical valve prostheses, cardiomyopathies, congenital heart disease, obstructive sleep apnoea syndrome (OSAS) and/or active malignancy. The study was approved by the Ethics Committee of the City of Health and Science of Turin (Approval n D15D18000410001). Of the 404 recruited patients, 307 were included in the study (Figure 1). All participants provided written informed consent. At the time of recruitment, data on demographic and clinical characteristics were collected, including age, sex, ethnicity, dietary habits, physical activity, alcohol consumption, smoking status, CV risk factors, family history of diabetes, duration of T2DM, comorbidities, chronic complications and current therapy. All participants underwent a comprehensive physical examination, and fasting blood samples were collected for biochemical analyses. Additionally, morning urine samples were obtained to measure the albumin‐to‐creatinine ratio (ACR). Further evaluations included a fundus oculi examination, a 12‐lead electrocardiogram (ECG), bioelectrical impedance analysis (BIA) and transthoracic echocardiography.

### 2.2 Biochemistry

Glycated haemoglobin (HbA1c) was measured using an immunoenzymatic assay, with values standardised according to the DCCT method. Blood glucose, total cholesterol, triglycerides, high‐density lipoprotein (HDL) cholesterol and serum creatinine levels were determined using standardised enzymatic methods on a Cobas‐Bio analyser. Urinary albumin and creatinine concentrations were measured using an immunoturbidimetric assay.

### 2.3 Measurements, Definitions and Calculations

Body mass index (BMI) was calculated as weight in kilogrammes divided by height in metres squared (kg/m^2^). Waist circumference (WC) was measured in the horizontal plane at the superior border of the right iliac crest at the end of a normal exhalation. Visceral obesity was defined as a WC ≥ 94 cm in men and ≥ 80 cm in women, according to the International Diabetes Federation (IDF) criteria [33]. Obesity was defined as a BMI ≥ 30 kg/m^2^. Neck circumference (NC) was measured with the participant standing upright and facing the examiner, with the head positioned according to the Frankfurt horizontal plane and the shoulders relaxed. A tailor's measuring tape was used, placed horizontally just below the laryngeal prominence and perpendicular to the long axis of the neck. NC was normalised by height (NCI = NC/height) to account for individual differences in body size [22, 24]. Only two trained operators performed all NC measurements, and the inter‐rater reliability was excellent (intraclass correlation coefficient [ICC] = 0.95). Body composition, including fat mass (FM), fat‐free mass (FFM) and skeletal muscle mass (SM), was estimated using bioelectrical impedance analysis (BIA) with the Akern BIA 101 device (Akern, Italy). Systolic and diastolic blood pressure (BP) was measured using a standard sphygmomanometer (Hawksley, Lancing, UK). Mean arterial blood pressure (MAP) was calculated using the formula: diastolic BP + (systolic BP − diastolic BP)/3. Hypertension was defined as a systolic BP ≥ 130 mmHg and/or a diastolic BP ≥ 80 mmHg, confirmed on at least two separate occasions, or current use of antihypertensive medication. Low‐density lipoprotein (LDL) cholesterol was calculated using the Friedewald formula.

OSAS was defined based on a previously established diagnosis. Patients identified as high‐risk through clinical assessment were excluded from the study if subsequent polysomnography confirmed the diagnosis. Participants were classified as albuminuric if they exhibited a urinary ACR ≥ 3 mg/mmol in at least two out of three measurements taken within a 6‐month period. Estimated glomerular filtration rate (eGFR) was calculated from serum creatinine levels using the CKD‐EPI equation. Chronic kidney disease (CKD) was defined as an eGFR ≤ 60 mL/min/1.73 m^2^. Diabetic retinopathy was assessed by a specialised ophthalmologist through the evaluation of retinal images obtained using the Optomed Aurora system (Midimedical). Retinopathy was classified as either absent or present (non‐proliferative or proliferative). For classification purposes, the eye with the more severe presentation was considered. Coronary artery disease (CAD) was defined as a history of myocardial infarction, angina pectoris, percutaneous coronary intervention (PCI), or coronary artery bypass grafting (CABG). Patients with clinical, electrocardiographic or echocardiographic findings suggestive of CAD were excluded from the study if further diagnostic investigations confirmed the presence of CAD.

### 2.4 Echocardiography

Transthoracic echocardiographic studies were performed using a Philips EPIQ CVx ultrasound system (Philips Healthcare, Andover, MA, United States) equipped with an X5‐1 matrix‐array transducer. All examinations were conducted by a cardiologist certified by the European Association of Cardiovascular Imaging (EACVI), following standardised protocols to ensure reproducibility and compliance with EACVI guidelines.

High‐resolution 2D images were acquired in standard parasternal, apical, and subcostal views, with frame rates optimised between 50 and 80 frames per second for dynamic imaging. Doppler techniques, including pulsed‐wave, continuous‐wave, colour Doppler, and tissue Doppler imaging (TDI), were employed to assess intracardiac flows, valvular regurgitation, and stenosis, with settings individually adjusted for flow velocities and anatomical landmarks. Echocardiographic image analysis was performed using QLab software (Philips Healthcare) integrated with TomTec Arena. Key structural and functional parameters of the left ventricle were measured, including left ventricular ejection fraction (LVEF), end‐diastolic diameter (EDD), end‐diastolic volume (EDV), end‐systolic volume (ESV), and septal and posterior wall thickness. Left ventricular mass (LVM) and relative wall thickness (RWT) were calculated. LVM was indexed to height raised to the power of 2.7 (LVMi, g/m^2.7^) to account for differences in body size [34]. LVH was defined as an LVMi ≥ 50 g/m^2.7^ in men and ≥ 47 g/m^2.7^ in women [35]. LVH was considered concentric if the RWT value was > 42.

### 2.5 Statistical Analysis

Results are presented as mean ± standard deviation (SD) for normally distributed variables, geometric mean with interquartile range (25th–75th percentile) for non‐normally distributed variables, and percentages for categorical variables. Normality was assessed using both the Shapiro–Wilk and Kolmogorov–Smirnov tests. Group comparisons were performed using two‐tailed Student's *t*‐tests, with logarithmic transformation of non‐normally distributed variables (e.g., ACR, triglycerides). Categorical variables were compared using the Chi‐square test. Pearson's correlation analysis was used to evaluate the correlation between LVMi, NCI and the other parameters. Logistic regression analyses were performed to assess whether NCI, BMI and WC were independently associated with the odds of LVH after adjusting for potential confounders. Analyses were performed both in the overall cohort and stratified by sex. To address multicollinearity between NCI, BMI, and WC, a residual regression approach was used. Residuals from models including either BMI or WC were computed and then regressed on NCI to assess its independent association with LVH. Receiver operating characteristic (ROC) curve analyses were performed to assess the performance of NCI in predicting LVH in men and women. For each analysis, the area under the curve (AUC) and the Youden index were calculated. A *p* value < 0.05 was considered statistically significant. Statistical analyses were performed using SPSS software version 29 (IBM Corp., Armonk, NY, USA).

## 3 Results

### 3.1 Study Population

Table 1 shows the demographic, anthropometric, and clinical characteristics of the 307 T2DM subjects recruited in the study. The study population had a mean age of 60.91 ± 8.32 years, with a slight predominance of male participants. Most participants were of Caucasian ethnicity (96.4%). More than half of the patients (56%) were obese and excess visceral adiposity was present in nearly all individuals (96.1%). While only 19.5% were active smokers, most (87.9%) reported low to moderate levels of physical activity. On average, patients had a relatively short duration of T2DM and exhibited good metabolic control. The prevalence of arterial hypertension, albuminuria, CKD, and diabetic retinopathy was 92.5%, 14.0%, 7.8% and 5.0%, respectively.

**TABLE 1:** Demographic and clinical characteristics of the 307 subjects with type 2 diabetes in the TESEO study.

Variables	Study population *n* = 307
Age (yrs)	60.91 ± 8.32
Male gender (%)	52.8
Caucasian ethnicity (%)	96.4
Smoking status (%)	
Never smokers	43.3
Former smokers	37.1
Active smokers	19.5
Physical activity (%)	
Low	37.1
Intermediate	50.8
High	12.0
Body mass index (Kg/m^2^)	31.24 ± 5.50
Waist circumference (cm)	109.62 ± 12.65
Fat mass (%)	17.21 ± 7.35
Diabetes duration (yrs)	3.76 ± 5.00
HbA1c (%)	6.51 ± 1.00
Systolic BP (mmHg)	136.99 ± 16.15
Diastolic BP (mmHg)	83.95 ± 9.60
MAP (mmHg)	101.63 ± 10.71
Total cholesterol (mg/dL)	175.70 ± 43.62
LDL‐cholesterol (mg/dL)	98.36 ± 36.12
HDL‐cholesterol (mg/dL)	51.56 ± 12.66
Triglycerides (mg/dL)	114.27 (85.0–146.0)
ACR (mg/mmol)	1.03 (0.50–1.58)
eGFR (ml/min/1.73 m^2^)	87.65 ± 16.22

*Note:* Data are expressed as mean ± SD, percentage or geometric mean (25°–75° percentile) for log transformed data.

Abbreviation: ACR, albumin‐creatinine ratio; BP, blood pressure; eGFR, estimated glomerular filtration rate; HDL, high‐density lipoprotein; LDL, low‐density lipoprotein; MAP, mean arterial pressure.

### 3.2 Left Ventricular Mass

The mean LVMi was 43.59 ± 11.35 g/m^2.7^ (42.57 ± 11.23 g/m^2.7^ in men and 44.72 ± 11.41 g/m^2.7^ in women). LVMi correlated directly and significantly with age, indices of adiposity (BMI, WC, FM, FFM), metabolic parameters (HbA1c, ln‐triglycerides), ln‐ACR and eGFR (Table 2).

**TABLE 2:** Pearson's correlation.

Variable	LVMi		NCI		*N*
	*r*	*p* value	*r*	*p* value	
Age	0.133	**0.019**	0.039	0.493	307
Systolic BP	0.087	0.127	0.130	**0.023**	307
Diastolic BP	0.076	0.185	0.153	**0.007**	307
MAP	0.089	0.119	0.157	**0.006**	307
Body mass index	0.417	**<** **0.001**	0.628	**<** **0.001**	307
Waist circumference	0.397	**<** **0.001**	0.615	**<** **0.001**	307
FFM	0.163	**0.006**	0.404	**<** **0.001**	278
FM	0.300	**<** **0.001**	0.450	**<** **0.001**	278
SM	−0.006	0.919	0.173	**0.004**	278
HbA1c	0.152	**0.008**	0.074	0.196	307
Ln‐triglycerides	0.170	**0.003**	0.112	0.05	307
HDL‐cholesterol	−0.063	0.272	−0.229	**<** **0.001**	307
eGFR	−0.132	**0.021**	−0.118	**0.039**	307
Ln‐ACR	0.127	**0.026**	0.091	0.112	307
Uric acid	0.095	0.109	0.250	**<** **0.001**	287

*Note:* Bold values indicate *p* value statistically significant.

Abbreviations: ACR, albumin‐creatinine ratio; BP, blood pressure; eGFR estimated glomerular filtration rate; FFM, fat‐free mass; FM, fat mass; HDL, high‐density lipoprotein; LVMi, left ventricular mass index; MAP, mean arterial pressure; NCI, neck circumference‐to‐height index; SM, skeletal muscle mass.

The overall prevalence of LVH was 29.3%, with a significantly higher rate in women (39.3%) than in men (20.4%; *p* < 0.001). Concentric LVH was the predominant geometric pattern (88.9%). Table 3 presents the demographic, anthropometric, clinical, and echocardiographic characteristics of patients stratified by LVH status. Compared with those without LVH, patients with LVH were older and had significantly higher HbA1c, triglycerides, BMI, WC, FM values and lower eGFR. Moreover, the prevalence of both obesity and visceral obesity was significantly greater in the LVH group. As expected, structural echocardiographic parameters of the left ventricle were significantly greater in patients with LVH, who were also more frequently treated with angiotensin‐converting enzyme (ACE) inhibitors or angiotensin II receptor blockers (ARBs).

**TABLE 3:** Demographic and clinical characteristics of the 307 subjects with type 2 diabetes in the TESEO study stratified by LVH status.

Variables	Non‐LVH *n* = 217	LVH *n* = 90	*p* value
Age (yrs)	60.22 ± 8.32	62.59 ± 8.12	**0.023**
Male gender (%)	59.4	36.7	< 0.001
Smoking status (%)			
Never smoker	42.4	45.6	0.685
Former smoker	38.7	33.3	
Active smoker	18.9	21.1	
Physical activity (%)			
Low	35.4	41.4	0.490
Intermediate	51.4	49.4	
High	13.2	19.2	
Body mass index (Kg/m^2^)	30.04 ± 5.21	34.13 ± 5.14	**<** **0.001**
Obesity (%)	45.6	81.1	**<** **0.001**
Waist circumference (cm)	107.39 ± 11.64	114.99 ± 13.40	**<** **0.001**
Visceral adiposity (%)	94.5	100.0	**0.021**
Fat mass (%)	15.86 ± 7.23	20.31 ± 6.68	**<** **0.001**
NC (cm)	39.74 ± 3.53	40.51 ± 3.73	**0.044**
NCI (cm/m)	23.85 ± 1.81	25.19 ± 1.80	**0.001**
Diabetes duration (yrs)	3.63 ± 4.95	4.07 ± 5.16	0.486
HbA1c (%)	6.42 ± 0.93	6.72 ± 1.12	**0.019**
Systolic BP (mmHg)	136.53 ± 15.89	138.12 ± 16.78	0.431
Diastolic BP (mmHg)	83.73 ± 9.03	84.05 ± 10.88	0.554
MAP (mmHg)	101.33 ± 10.24	102.37 ± 11.80	0.464
Hypertension (%)	91.7	94.4	0.483
Total cholesterol (mg/dL)	177.0 ± 44.0	172.6 ± 42.8	0.423
LDL‐cholesterol (mg/dL)	100.0 ± 35.4	94.4 ± 37.6	0.218
HDL‐cholesterol (mg/dL)	51.8 ± 13.0	51.0 ± 11.9	0.649
Triglycerides (mg/dL)	110.00 (82.0–144.0)	125.25 (99.8–147.8)	**0.008**
ACR (mg/mmol)	0.98 (0.49–1.40)	1.17 (0.60–2.03)	0.239
Albuminuria (%)	14.3	13.3	0.859
eGFR (ml/min/1.73 m^2^)	89.08 ± 15.64	84.21 ± 17.14	**0.016**
CKD (%)	7.4	8.9	0.816
LVM (g)	151.24 ± 33.71	208.93 ± 45.21	**<** **0.001**
LVMi (g/m^2.7^)	37.81 ± 6.27	57.51 ± 8.39	**<** **0.001**
IVS (mm)	10.48 ± 1.36	11.84 ± 1.12	**<** **0.001**
PW (mm)	9.84 ± 1.15	11.38 ± 1.18	**<** **0.001**
RWT (mm)	0.45 ± 0.07	0.48 ± 0.07	**0.001**
RWT > 0.42 (%)	71.4	88.9	**<** **0.001**
EF (%)	61.24 ± 4.09	61.53 ± 3.80	0.565
SGLT2 inhibitors	21.7	17.8	0.535
GLP1‐RA	28.1	31.1	0.679
ACE inhibitors/ARB	46.5	61.1	**0.024**

*Note:* Data are expressed as mean ± SD, percentage or geometric mean (25°–75° percentile) for log transformed data. Bold values indicate *p* value statistically significant.

Abbreviations: ACE, Angiotensin‐Converting Enzyme; ACR, albumin‐creatinine ratio; ARB, Angiotensin II receptor blockers; BP, blood pressure; CKD chronic kidney disease; EF, ejection fraction; eGFR, estimated glomerular filtration rate; GLP1‐RA, Glucagon‐like peptide‐1 receptor agonists; HDL, high‐density lipoprotein; IVS, interventricular septum; LDL, low‐density lipoprotein; LVH, left ventricular hypertrophy; LVM, left ventricular mass; LVMi, left ventricular mass index; PW, posterior wall; RWT, relative wall thickness; SGLT2 Sodium‐glucose co‐transporter 2.

### 3.3 NC and NCI

In all cohorts, mean NC values were 39.96 ± 3.60 (women: 37.72 ± 2.74; men: 41.97 ± 3.06). The average NCI values were 24.24 ± 1.90 (women: 24.02 ± 1.89; men: 24.44 ± 1.90). As shown in Table 2, NCI values showed significant correlations with indices of adiposity (BMI, WC), dysmetabolism (HDL‐cholesterol, systolic BP, diastolic BP, MAP, uric acid), and renal dysfunction (eGFR). In bioelectrical impedance analysis, NCI correlated positively with fat mass, fat‐free mass, and skeletal muscle mass, likely reflecting the inability of NC measurement to distinguish fat from lean tissue and the known link between adiposity and greater muscle mass due to mechanical load.

Subjects with LVH had significantly higher NC and NCI values. This was also confirmed when the analysis was performed separately in men (NC: 44.37 ± 3.55 vs. 41.66 ± 2.89 cm, *p* < 0.001; NCI: 26.08 ± 1.98 vs. 24.43 ± 1.76 cm/m, *p* < 0.001) and women (NC: 39.06 ± 2.70 vs. 37.28 ± 2.97 cm, *p* < 0.001; NCI: 25.15 ± 1.89 vs. 23.57 ± 1.96 cm/m, *p* < 0.001).

### 3.4 Logistic Regression Analyses

Logistic regression analysis was conducted to evaluate whether NCI was independently associated with an increased odds of LVH, adjusting for potential confounders and established risk factors (Table 4). In the overall population, each 1 standard deviation (SD) increase in NCI was significantly associated with a 1.57‐fold higher OR of LVH, independent of age, sex, HbA1c, ln‐triglycerides, eGFR, ln‐ACR, MAP, and treatment with SGLT2 inhibitors, GLP1‐RA, and ACE inhibitors/ARB. When analyses were stratified by sex, the association remained significant in both men and women, but was stronger in men than in women. Similar results were obtained when NC was used in the analyses, though the strength of the associations was lower.

**TABLE 4:** Adjusted odds ratios (ORs) for LVH in the overall population by sex and for residualized NCI models.

Model	Independent variable	Overall OR (95% CI)	Men OR (95% CI)	Women OR (95% CI)
Standard model	NC	1.259 (1.128–1.406)	1.275 (1.078–1.508)	1.259 (1.074–1.475)
	NCI	1.567 (1.322–1.858)	1.649 (1.246–2.181)	1.491 (1.188–1.871)
	BMI	1.166 (1.097–1.240)	1.215 (1.094–1.349)	1.132 (1.047–1.224)
	WC	1.061 (1.034–1.089)	1.073 (1.031–1.117)	1.047 (1.011–1.084)
Residualized NCI	NCI residualized on BMI (NCI‐BMI)	1.354 (1.098–1.670)		
	NCI residualized on WC (NCI‐WC)	1.416 (1.167–1.719)		

*Note:* Adjusted for age, sex, HbA1c, ln‐transformed triglycerides (lnTG), estimated glomerular filtration rate (eGFR), ln‐transformed albumin‐to‐creatinine ratio (lnACR), uric acid, mean arterial pressure (MAP), antihyperglycemic drugs (SGLT2 inhibitors, GLP‐1 receptor agonists) and antihypertensive drugs (ACE inhibitors/ARBs). In analyses stratified by sex, sex was not included as a covariate.

Abbreviations: BMI, body mass index; CI, confidence interval; NC, neck circumference; NCI, neck circumference index; OR, odds ratio; WC, waist circumference.

BMI and WC were not included in the model because of multicollinearity. However, when NCI was replaced by BMI in the model, the strength of the association with LVH was markedly reduced (OR 1.17 [95% CI 1.10–1.24]) and further diminished when NCI was replaced by WC (OR 1.06 [95% CI 1.03–1.09]). We also applied the residual method by regressing NCI on BMI and WC and using the unstandardised residuals in the model. The residualized NCI remained significantly associated with increased odds of LVH (NCI‐BMI residual OR 1.35 [95% CI 1.10–1.67]; NCI–WC residual OR 1.42 [95% CI 1.17–1.72]; Table 4), indicating that NCI captures unique variance in LVH beyond that explained by BMI and WC.

### 3.5 ROC Curve Analyses

Receiver operating characteristic (ROC) curve analyses were conducted to assess the performance of NCI in predicting LVH in men and women. The area under the curve (AUC) was 0.711 for women and 0.739 for men, indicating good discriminatory performance. The optimal NCI cut‐off values, identified using the Youden index, were 23.47 for women and 25.14 for men.

## 4 Discussion

This study provides the first evidence that both NC and NCI are independently associated with LVH in a cohort of patients with T2DM.

Mean NC values exceeded the established normal thresholds (NC < 37 cm in men and < 34 cm in women) [36], consistent with findings from previous studies in both obese and T2DM subjects [37, 38]. Absolute NC values were normalised to height to derive the NCI, which more accurately reflects body shape and upper‐body adiposity [28].

NCI values were significantly higher in individuals with LVH. Furthermore, logistic regression analysis revealed that elevated NCI was associated with a 57% increased OR of LVH, independent of age, sex, MAP, HbA1c, triglycerides, eGFR, ACR, and treatment with SGLT2 inhibitors, GLP1‐RA, and ACE inhibitors/ARB. Extending the well‐established association between adiposity indices and LVH [39, 40], these findings highlight NCI as a relevant anthropometric marker for LVH. Consistently, ROC curve analyses confirmed that NCI has good discriminatory power for identifying individuals with and without LVH, and revealed sex‐specific optimal cut‐off values that may be clinically useful for early LVH screening in patients with T2DM.

The mechanisms linking adiposity to LVH remain incompletely understood; however, obesity is thought to contribute to LVH development through chronic haemodynamic overload, elevated free fatty acid (FFA) release and low‐grade systemic inflammation [41]. In our study, although blood pressure correlated with NCI, patients with and without LVH did not differ in blood pressure. Furthermore, the association between NC/NCI and LVH persisted after adjustment for MAP and treatment with ACE inhibitors/ARBs, suggesting that blood pressure is unlikely to play a major role in this relationship. Importantly, NCI is considered a proxy for upper‐body subcutaneous fat (UBSF), a major source of circulating FFAs in obese individuals [42], which can also secrete proinflammatory cytokines, including tumour necrosis factor‐α and interleukin‐6 [43]. Whether FFAs and low‐grade inflammation mediate the relationship between NC/NCI and LVH remains unknown and this hypothesis needs to be tested in future studies.

In our cohort, LVH prevalence was 29.3%, predominantly exhibiting a geometric remodelling pattern, consistent with previous reports in T2DM patients [39]. LVH was nearly twice as common in women compared to men, aligning with findings from the Framingham Heart and Casale Monferrato studies [44, 45]. Although the underlying mechanisms remain unclear, sex differences in myocardial function, potentially influenced by hormonal factors, may be amplified in women with T2DM, contributing to this disparity. Notably, sex differences were also evident in the association between NCI and LVH. Logistic regression stratified by sex showed that NCI increased the odds of LVH by 65% in men and 49% in women. Consistently, a longitudinal study showed that NC was a better predictor of future CV events in men than in women [30]. Men tend to accumulate more UBSF than women; therefore, NCI might better capture the adverse fat‐related effects on the heart in men compared with women. Additionally, hormonal differences, such as higher testosterone levels in men, could also influence fat deposition patterns and cardiac remodelling [31, 46].

Individuals with LVH exhibited higher BMI and WC values, along with a greater prevalence of obesity and visceral adiposity. Due to concerns about collinearity, BMI and WC were not included in the logistic regression model. Nonetheless, the adjusted associations between LVH and both BMI and WC were weaker than those observed with NCI, suggesting a more prominent role for NCI compared to traditional anthropometric measures. Additionally, residual analyses revealed that NCI explained an additional ∼37% of the variance in LVH beyond that accounted for by BMI and WC.

This study has several limitations. First, its cross‐sectional design limits our ability to infer causal relationships between NC/NCI and LVH. The observed association may reflect a causal effect, a consequence, or the influence of common underlying factors. Nonetheless, no previous data are available in a large T2DM cohort, and our findings may serve as a valuable starting point for further investigation. Second, all participants were recruited from a single Diabetes Centre in Turin, Italy, and were predominantly Caucasian. As NC/NCI thresholds and body composition may differ across ethnic groups, this limits the generalisability of our findings and further studies in more diverse cohorts are needed to confirm their external validity. Third, the prevalence of LVH in our cohort was lower than that reported in previous studies [45, 47], likely reflecting the shorter diabetes duration and better glycaemic control of our participants. Indeed, similar LVH prevalence rates have been observed in cohorts with comparable clinical profiles [48, 49]. Finally, sleep studies were not systematically performed prior to inclusion to rule out undiagnosed cases of OSAS; however, participants identified as high‐risk based on clinical assessment were excluded if subsequent polysomnography confirmed the diagnosis.

The study also has several strengths. First, it is based on a relatively large cohort of patients with T2DM. Second, individuals with known cardiac or thyroid diseases were excluded, thereby minimising potential confounding. Third, the analysis was conducted on a contemporaneous cohort, reflecting current clinical profiles and risk factor distributions, which enhances the relevance of the findings to present‐day clinical practice. Moreover, medications known to influence both LVH progression and blood pressure—ACE inhibitors/ARBs, SGLT2 inhibitors, GLP1‐RA—were included in the logistic regression models to account for potential residual confounding. Finally, LVH was diagnosed using echocardiography, which offers greater accuracy than electrocardiography, the method employed in several previous studies investigating the association between anthropometric measures and LVH.

In conclusion, our study highlights the potential value of the NCI as a novel anthropometric marker associated with LVH in patients with T2DM. The findings suggest that NCI may offer additional information beyond traditional anthropometric parameters. However, prospective longitudinal studies are needed to establish whether NCI can reliably identify individuals at increased risk of developing LVH.

## Author Contributions

F.B. manuscript writing, data analysis and interpretation. A.A. manuscript writing, data collection and interpretation. M.B., G.B., A.F., M.B., S.B., G.M. data collection. G.M., G.A., F.B., G.M.D.F. manuscript reviewing and editing. G.G. study conception and design, manuscript writing, data interpretation. G.G. is the guarantor of this work and, as such, had full access to all the data and takes responsibility for the integrity of the data and accuracy of the data analysis.

## Conflicts of Interest

The authors declare no conflicts of interest.

## Peer Review

The peer review history for this article is available at https://www.webofscience.com/api/gateway/wos/peer-review/10.1002/dmrr.70106.

## Data Availability

The data that support the findings of this study are available from the corresponding author upon reasonable request.
